# Targeting Osteoporosis-Osteoarthritis Comorbidity: Multi-Omics Identification of SON and Exploration of Therapeutic Agents

**DOI:** 10.3390/ijms27114905

**Published:** 2026-05-28

**Authors:** Kunlong Jiang, Jun Du, Peng Yang, Liyun Lin, Jiachen Liu, Yusen Qiao, Huilin Yang, Dechun Geng, Kun Li

**Affiliations:** 1 Department of Orthopedic Surgery, The First Affiliated Hospital of Soochow University, No. 899 Pinghai Road, Suzhou 215000, China; jiangkunlong@163.com (K.J.);; 2 Department of Orthopedic Magnetic Resonance Chamber, The First Affiliated Hospital of Soochow University, No. 899 Pinghai Road, Suzhou 215000, China

**Keywords:** osteoporosis, osteoarthritis, targeted therapy

## Abstract

Osteoporosis (OP) and osteoarthritis (OA) frequently coexist in the elderly, yet shared molecular pathways remain unclear. We employed weighted gene co-expression network analysis (WGCNA), independent cohort differential verification, and integration of multiple datasets to explore the association between the two diseases and identify the hub genes. Then, through Mendelian randomization and single-cell sequencing methods, we confirmed that SON might be the key protein linking these two diseases and a promising therapeutic target for comorbidity. Additionally, this study utilized the FDA drug database for virtual screening to evaluate the potential of SON protein as a drug target. Nilotinib, with a high docking score, was listed as a candidate drug. Overall, our findings suggest that SON may play a protective role in the comorbidity of OP and OA, but further functional studies are required to confirm its causal role. Moreover, Nilotinib shows inhibitory effects on osteoclast differentiation and targets SON in vitro, indicating its potential as a candidate drug for further investigation.

## 1. Introduction

Elderly individuals often present with multimorbid musculoskeletal disorders [1], including osteoporosis (OP) and osteoarthritis (OA), in addition to a single disease. Aging serves as a bridge between OP and OA, facilitating the coexistence of these conditions, which is particularly common among the elderly. The advancement of this comorbidity not only amplifies the intricacy of therapeutic interventions but also subjects patients to elevated health risks. OP is typically characterized by a systemic reduction in bone mineral density (BMD) and thinning of the bone structure, while OA is characterized by the degeneration of articular cartilage and osteosclerosis resulting from increased BMD. Despite clinical and etiologic differences, both disorders involve musculoskeletal abnormalities and share several pathogenic mechanisms.

Currently, randomized clinical trials and meta-analyses have found that the relationship between OP and OA remains uncertain, and the exact mechanisms explaining the coexistence of these two diseases are still unclear. And the advancement of bioinformatics and the application of gene chips in recent years have turned bioinformatics analysis into an indispensable tool in the medical field [2]. Therefore, this study aims to explore the common characteristics of these diseases through multi-omics analysis, providing a new perspective for the treatment and prevention of the diseases.

We extensively collected sequencing data related to OP and OA from the Gene Expression Omnibus (GEO) database. To connect the modules to each other and to external clinical features, a weighted gene co-expression network analysis (WGCNA) approach was used. The expression profiles of hub genes in patients with OP and OA were validated by single-cell sequencing data [3]. In addition, we investigated the transcriptional regulation of hub genes and used the database to predict the relevant miRNAs. Genome-Wide Association Studies (GWAS) have played a crucial role in investigating complex traits, while Mendelian Randomization (MR), based on Mendel’s laws, served as a genetic method for causal inference. Leveraging single nucleotide polymorphisms (SNPs) as instrumental variables [4], MR allows us to elucidate causal relationships between observed phenotypic exposures and genetic expression outcomes. Finally, we further verified the expression and function of hub genes in single-cell sequencing and in vitro cell experiments and obtained relevant therapeutic drugs through virtual screening.

In this study, we integrated different databases and employed multi-omics analysis to systematically unveil the genetic association between OP and OA at the genetic level. Our objective is to identify genes co-expressed in both diseases, uncover their causal relationships in disease pathogenesis, and find suitable drugs that can provide new assistance to patients with osteoporosis and osteoarthritis.

## 2. Results

### 2.1. Co-Expression Network Construction and Hub Module Identification

**Figure 1:**
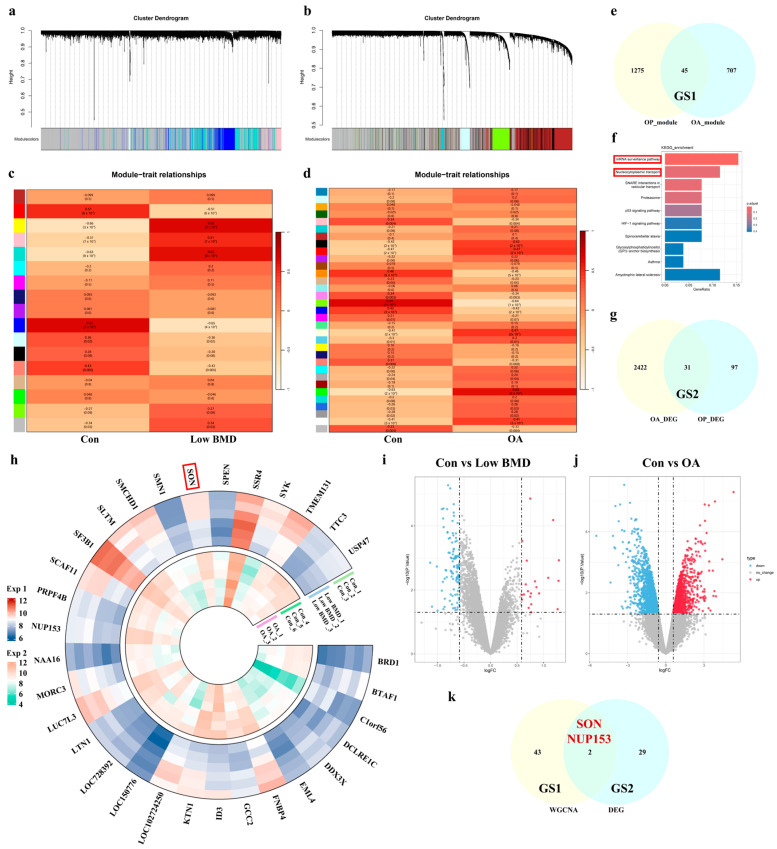
Weighted gene co-expression network analysis (WGCNA) and differentially expressed gene (DEG) analysis results from validation cohort. (**a**) The cluster dendrogram of co-expression genes in Low BMD. (**b**) The cluster dendrogram of co-expression genes in OA. (**c**) Module–trait relationships in Low BMD. Each cell contains the corresponding correlation coefficient and *p*-value. (**d**) Module–trait relationships in OA. Each cell contains the corresponding correlation coefficient and *p*-value. (**e**) Venn diagram showing the intersection of genes. (**f**) KEGG pathway analysis. Con, control group. OA, the group of osteoarthritis. Low BMD, the group with low bone mineral density. (**g**) Venn diagram determines the common DEGs, referred to as GS2. (**h**) Heatmap of GS2. Red indicates upregulated genes, and blue or green indicates downregulated genes. (**i**) Volcano plot of DEGs in OP. Blue indicates downregulated DEGs, and red indicates upregulated DEGs. (**j**) Volcano plot of DEGs in OA. Blue indicates downregulated DEGs, and red indicates upregulated DEGs. (**k**) Venn diagrams showing common genes across different analysis methods and datasets.

We conducted WGCNA across multiple datasets to investigate common genetic features and molecular mechanisms underlying OP and OA. In the GSE56815 dataset, WGCNA identified different gene modules, represented by different colors (Figure 1a,b). Spearman’s module-trait graph showed that two modules, “yellow” and “turquoise” (Figure 1c), were strongly associated with Low BMD (yellow module: r = 0.66, *p* = 3 × 10^−6^; turquoise module: r = 0.63, *p* = 2 × 10^−9^), containing 543 genes and 777 genes, respectively (1320 genes in total). In the GSE48556 dataset, the “green” module showed a high association with OA and was selected as the osteoarthritis-related module (r = 0.63, *p* = 0.046), comprising 752 genes (Figure 1d). An intersection analysis of the module genes associated with Low BMD and OA identified 45 common genes (Figure 1e), which were grouped into Gene Set 1 (GS1, Table S1). To confirm the function of this gene set, we conducted a KEGG pathway analysis (Figure 1f). The results showed that GS1 mainly regulates the mRNA surveillance pathway and Nucleocytoplasmic transport.

To further verify disease specificity in the validation cohort, public datasets were preprocessed and thoroughly quality-controlled via principal component analysis (PCA) and cluster analysis (Figure S1). We used the GSE7429 dataset as a validation cohort to identify DEGs between Low-BMD and normal-BMD samples using the limma-R packages (v3.56.2). A total of 128 DEGs were identified using *p* < 0.05 and |logFC| > 0.585 as the screening criteria (Figure 1i). Similarly, analysis of the GSE12021 dataset identified 2453 DEGs associated with OA (Figure 1j). The intersection of DEGs from both datasets identified 31 common genes (Gene Set 2; GS2), the full list of which is detailed in Table S2 (Figure 1g). In the OP dataset (GSE56815), *SON* was significantly downregulated in the Low BMD group (log2FC = −0.76, *p* = 6.71 × 10^−6^); in the OA dataset (GSE48556), *SON* was also significantly downregulated in the OA group (log2FC = −0.92, *p* = 0.0092). The gene expression heatmap of GS2 is shown in Figure 1h. A further intersection of GS2 and GS1 identified two overlapping genes, *SON* and *NUP153*, which were identified as the hub genes for subsequent analysis (Figure 1k). To further characterize the features of OP and OA, we integrated additional datasets encompassing low peak bone mass (PBM) and articular cartilage profiles (GSE7158, GSE114007). These additional analyses underscore the pivotal role of *SON*, which exhibited significant downregulation in both the low PBM cohort (log2FC = −0.62, *p* = 0.0048) and the OA cohort (log2FC = −11.54, *p* = 1.85 × 10^−5^). This consistent suppressive pattern is further corroborated by volcano plots and a heatmap provided in Figure S2.

### 2.2. Causal Relationship Between Gene Expression and Phenotype Through MR

**Figure 2:**
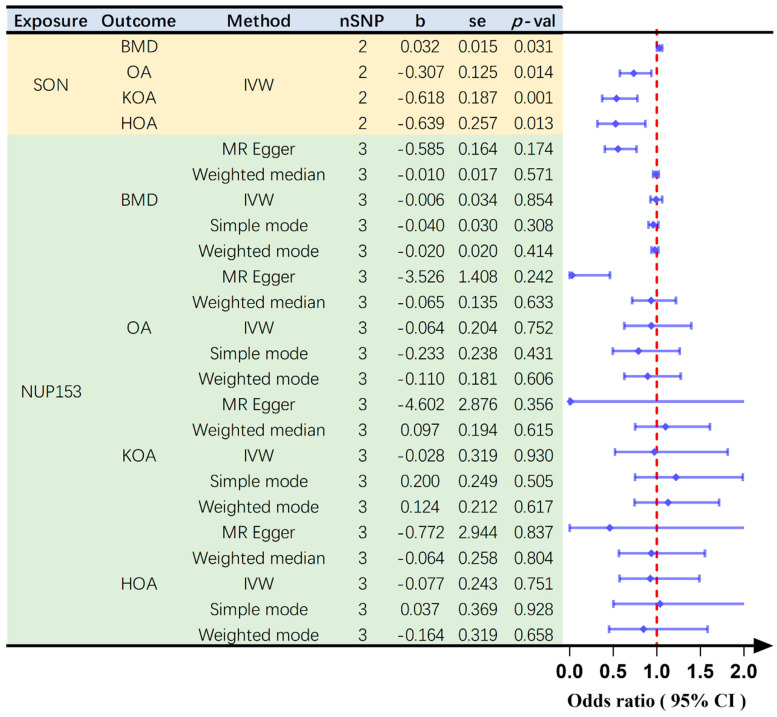
Causal relationship between hub genes and BMD or OA. Forest plot showing the impact of hub genes on BMD and OA. Abbreviations: BMD, bone mineral density; OA, osteoarthritis; KOA, knee osteoarthritis; HOA, hip osteoarthritis; CI, confidence interval.

We performed comprehensive MR analysis using the genes in GS1 as exposure factors and disease status as outcomes. The instrumental variables employed in the MR analysis are documented in Table S3. Comprehensive results concerning gene expression, BMD and osteoarthritis in GS1 are synthesized in Tables S4–S8. The MR analysis revealed a significant causal relationship between *SON* gene expression and BMD and OA (Figure 2), while no significant causal relationship was observed for *NUP153* (*p* > 0.05). Specifically, *SON* gene expression was positively correlated with BMD and negatively correlated with OA, showing a stronger association with knee osteoarthritis (KOA) and hip osteoarthritis (HOA). Conversely, reverse MR analysis, using BMD and OA as exposure and hub-gene expression as outcomes, did not yield a causal association. Results of the heterogeneity and pleiotropy test are shown in Table S9 and Figure S3. This study follows the Guidelines for Strengthening the Reporting of Mendelian Randomization Studies (STROBE-MR) checklist [5] to improve the interpretation of our results (Table S10, STROBE Statement).

### 2.3. Hub Gene Expression in Single Cells

**Figure 3:**
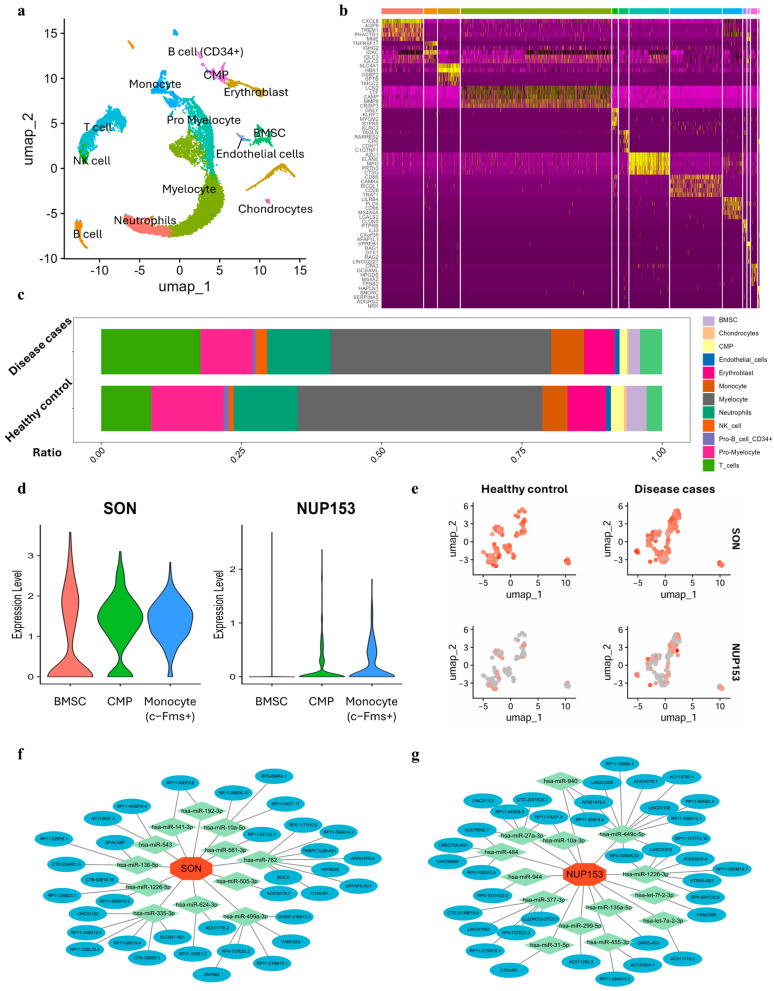
Expression profiles of hub genes in single cells and regulatory network of hub genes. (**a**) The UMAP plot shows color-coded clustering of human single-cell sequencing data across different groups. (**b**) Heatmaps show the top 5 maker genes expressed in each cell cluster. (**c**) Expression levels of hub genes in different cell clusters. (**d**) Relative expression of *SON* and *NUP153* in bone marrow cells. (**e**) Proportion of high *SON* expression (Expression level > 1) or *NUP153* high expression and c−Fms+ monocyte population in each group. (**f**,**g**) Regulatory network of *SON* and *NUP153*. Red represents mRNAs, green represents miRNAs, and blue represents lncRNAs.

We analyzed single-cell data from the GSE169396 dataset using the Seurat-R package (v5.1.0). The UMAP algorithm was used to cluster the cells (Figure 3a and Figure S4a–d), and each cluster was manually annotated into 13 cell types (Figure S4e,f and Table S11). The heatmap showing the top 5 maker genes expressed in each cell cluster is presented in Figure 3b. Cell proportions across groups are depicted in Figure 3c. Compared with the control group, the proportion of BMSCs in the disease group was significantly lower. The proportion of monocytes, B cells and T cells went up. We extracted c−Fms+ cells (highly expressing CSF1R) from monocytes as osteoclast precursors (Figure S4g), and further analysis showed that *SON* was highly expressed in CMP and monocytes (c−Fms+), while relatively low in BMSC (Figure 3d). In addition, the disease group showed lower proportion of high *SON* expression; *NUP153* is less expressed in these cells (Figure 3e and Figure S5a). Through further temporal sequence analysis, the main subtypes of monocytes were divided into two stages, highlighting that bone marrow cells may have different functional states. Under pseudo-time, the expression level of *SON* and *NUP153* decreases over time, while the expression of osteoclast-specific genes (*NFATC1*, *MMP9*) increases (Figure S5b–d). These results indicate that *SON* is mainly expressed in monocytes, and its expression decreases in the comorbidity group.

### 2.4. Competing Endogenous RNA (ceRNA) Regulatory Network

KEGG analysis highlighted the role of mRNA surveillance pathways. We constructed a ceRNA network for the two hub genes using Cytoscape, based on data from TargetScan, miRanda, and miRDB (Figure 3f,g). These two genes were found to interact with five miRNAs, including hsa-miR-548n, hsa-miR-3163, hsa-miR-3143, hsa-miR-3119, and hsa-miR-302c-5p. To explore the regulatory role of long non-coding RNAs (lncRNAs) on these miRNAs, we used SpongeScan [6] to predict relevant interactions. Key lncRNAs were identified, which may regulate these miRNAs, offering insights into the comorbidity mechanisms of OP and OA.

### 2.5. The Expression of SON Decreases During rhRANKL-Induced Osteoclastogenesis In Vitro

**Figure 4:**
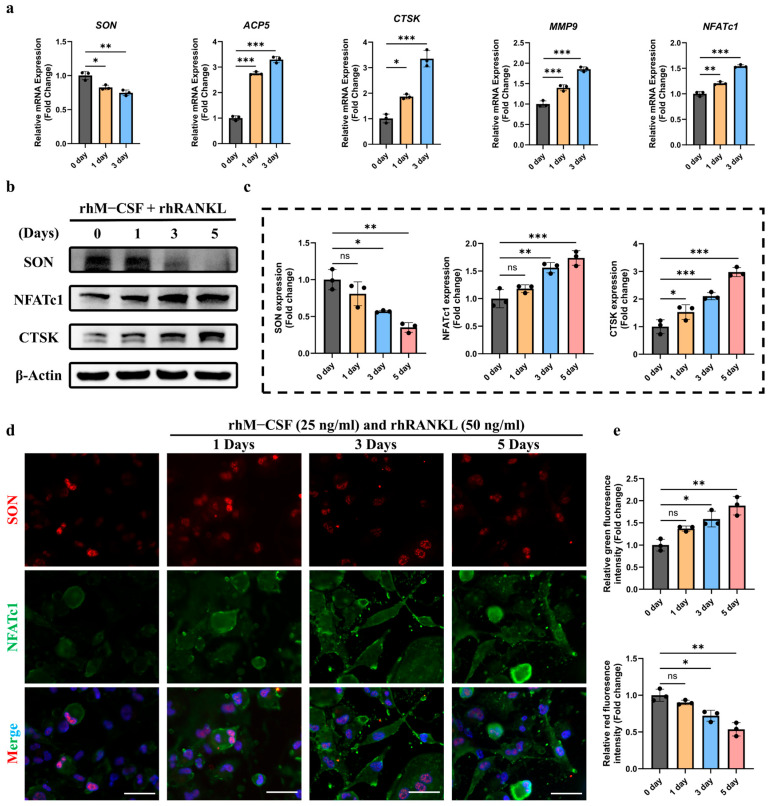
The expression of *SON* decreases during osteoclast differentiation. (**a**) The mRNA expression levels of *SON*, *ACP5*, *Cathepsin K*, *MMP9* and *NFATc1* in THP-1 by q-PCR (*n* = 3). (**b**) Western blot analysis of SON, NFATc1 and CTSK expression in osteoclasts cultured with rhM-CSF and rhRANKL. (**c**) The quantitation of the ratios of SON, NFATc1 and CTSK related to β-actin (*n* = 3). (**d**) Representative images of immunofluorescence staining show the activities of SON and NFATc1 in THP-1 cells induced by rhM-CSF and rhRANKL. (Scale bar  =  50 μm). SON (red), NFATc1 (green) and nuclei (DAPI, blue). (**e**) Quantitative analysis of the fluorescence intensity of SON, NFATc1 (*n* = 3). All experiments were repeated at least three times, and each group was compared with the group of 0 days. * *p* < 0.05, ** *p* < 0.01, *** *p* < 0.001, and ns: no significance. All data are presented as mean ± SD.

To further explore the effect of *SON* on osteoclast differentiation, we induced osteoclast differentiation in vitro using THP1 cells and measured the expression levels of *SON* at the mRNA and protein levels. THP-1 cells were first primed with PMA to induce adherence, then treated with rhM-CSF and rhRANKL for 1, 3, or 5 days to observe the changes in *SON* during osteoclastogenesis. Quantitative RT-PCR (Figure 4a) and Western blot analyses (Figure 4b,c) showed that the expression of osteoclast marker genes increased over time, such as *ACP5*, *CTSK*, *MMP9* and *NFATc1*. However, as the induction time of rhRANKL increases, the expression level of *SON* decreases. Similarly, immunofluorescence staining revealed that the expression of SON was inhibited during osteoclast differentiation, while enhancing the nuclear accumulation of NFATc1 (Figure 4d). Collectively, these results demonstrate that activation of osteoclast leads to a decrease in SON expression.

### 2.6. Potential Drug Prediction and Functional Identification

**Figure 5:**
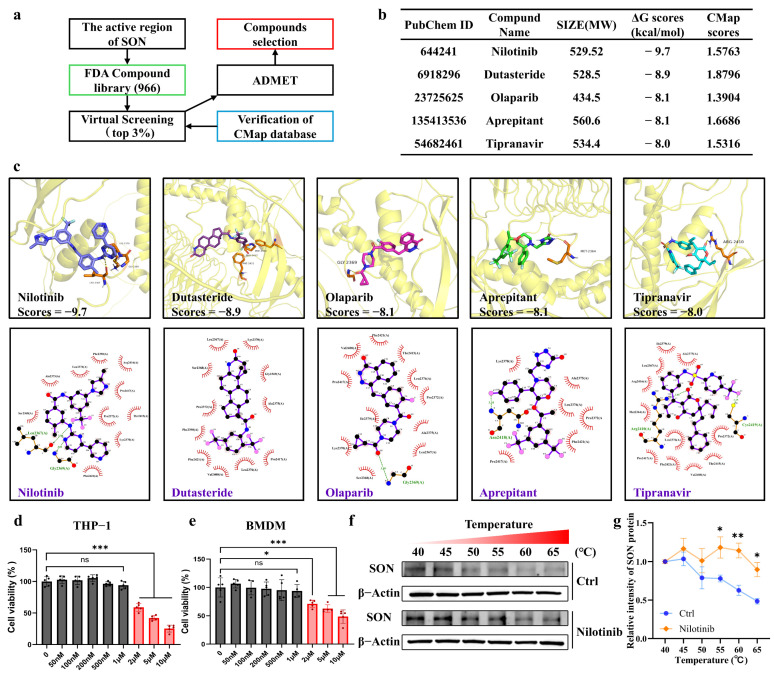
Virtual screening targeting the SON active site. (**a**) Flowchart of target-drug screening based on the SON active site. (**b**) Top five compounds ranked by molecular docking scores. (**c**) 3D and 2D diagrams showing hydrogen-bond interactions between SON residues and any of the following drugs: Nilotinib, Dutasteride, Olaparib, Aprepitant, or Tipranavir. (**d**) CCK-8 profiling of THP-1 viability following 72 h exposure to escalating Nilotinib doses (*n* = 5). (**e**) CCK-8 profiling of BMDMs viability following 72 h exposure to escalating Nilotinib doses (*n* = 5). (**f**) Thermal stability of SON protein in the presence or absence of Nilotinib treatment. (**g**) Quantitative statistics of SON expression normalized to β-actin expression in (**f**) (*n* = 3). * *p* < 0.05, ** *p* < 0.01, *** *p* < 0.001, and ns: no significance. All data are presented as mean ± SD.

To further explore the possibility of SON as a drug target, we conducted virtual screening using the FDA database. The process of finding drugs based on protein targets is shown in Figure 5a. Through molecular docking analysis, we identified five drugs with high affinity for SON, including Nilotinib, Dutasteride, Olaparib, Aprepitant, and Tipranavir (Figure 5b). Detailed results, including CMap scores and ADMET profiles for the top 3% of candidate drugs based on docking scores, are provided in Table S12. The results indicate that Nilotinib has a strong binding affinity (−9.7 kcal/mol) and can interact with PHE-2390, PRO-2417, and PHE-2421 of the SON protein (Figure 5c). In vitro experiments revealed that Nilotinib at concentrations below 1 μM showed no toxicity to THP1 cells and BMDMs (Figure 5d,e and Figure S6). The cellularthermal shift results indicated that Nilotinib could protect the thermal stability of the SON protein (Figure 5f,g). These results highlight the drug-targeting potential of the SON protein, indicating that by applying drugs such as Nilotinib, a spontaneous interaction with the target can occur, and there is the potential to limit the development of complications such as osteoporosis and osteoarthritis.

### 2.7. Nilotinib Inhibits RANKL-Induced Osteoclastogenesis In Vitro

**Figure 6:**
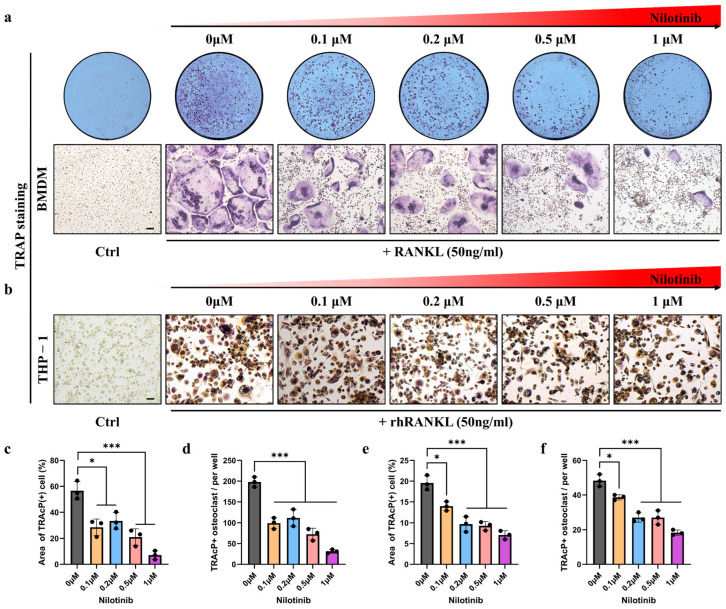
Attenuation of RANKL-stimulated osteoclast differentiation by Nilotinib. (**a**) Representative images of TRAP staining of osteoclast differentiation in BMDMs following Nilotinib treatment at indicated concentrations (scale bar = 100 μm). (**b**) Representative images of TRAP staining of osteoclast differentiation in THP-1 cells following Nilotinib treatment at indicated concentrations (scale bar = 100 μm). (**c**,**d**) Quantification of TRAP-positive multinucleated cells (nuclei > 3) per field and the average area of osteoclasts per group following Nilotinib treatment in (**a**). (**e**,**f**) Quantification of TRAP-positive multinucleated cells (nuclei > 3) per field and the average area of osteoclasts per group following Nilotinib treatment in (**b**). Bar graphs represent the mean ± SD, *n* = 3. * *p* < 0.05, *** *p* < 0.001, as compared to the positive control group.

To evaluate nilotinib’s therapeutic potential in osteoclastogenesis, we performed in vitro experiments using BMDM and THP-1 cells, with osteoclastogenesis induced by RANKL. RANKL stimulation in the control group generated abundant osteoclasts, whereas nilotinib treatment markedly suppressed osteoclastogenesis (Figure 6a,b). Nilotinib treatment suppressed osteoclastogenesis in a dose-dependent manner, with concentrations of 0.5–1 μM producing the most substantial reductions in osteoclast size and number (Figure 6c–f).

## 3. Discussion

The coexistence of OP and OA is increasingly observed in clinical practice, imposing significant economic and quality-of-life burdens on patients. The association between OP and OA has always been a topic of great interest [7,8,9]. These results align with previous studies in this field [10], highlighting the role of factors such as weight, aging [11], chronic inflammation [12], and decreased physical activity [13] in elevating OA risk.

To explore the shared molecular mechanisms between these two conditions, Through WGCNA, we identified gene modules associated with Low BMD and OA. Through subsequent analysis, we identified 31 common DEGs, and after cross-referencing with GS1, we pinpointed two hub genes: *SON* and *NUP153*. *SON* is a nuclear speck protein that binds DNA and RNA, playing roles in RNA splicing, gene transcription, cell cycle regulation, and the maintenance of embryonic stem cell [14,15,16]. It has been implicated in Zhu–Tokita–Takenouchi–Kim (ZTTK) syndrome, a condition characterized by developmental delays and musculoskeletal abnormalities due to heterozygous loss of *SON* function [17,18]. Furthermore, *SON* regulates RNA methylation and suppresses inflammation. In the context of OP and OA comorbidities, *SON* gene deletion may disrupt RNA splicing and, in turn, affect the metabolism and degradation of bone and joint tissues, contributing to disease progression. The KEGG pathway analysis also confirmed that hub genes are closely related to mRNA surveillance pathways [19].

To further substantiate these findings, we applied MR to show that *SON* expression is positively correlated with BMD and negatively correlated with OA, with stronger associations observed in knee and hip osteoarthritis. Additionally, in patients with OP and OA, the activation of subchondral bone osteoclasts plays a significant role in the deterioration of the disease [20,21]. And single-cell sequencing revealed the differential expression of hub genes among different cell types. The proportion of immune-related cells (e.g., B cells, T cells, and monocytes) was higher in the patient group than in the control group, reinforcing the critical role of immune response and inflammation in OP and OA comorbidity [22,23,24,25]. The results of single-cell analysis further indicate that the *SON* gene is widely expressed in the monocyte/macrophage cluster, and its expression level is significantly reduced in patients with comorbidity. Integrating pseudo-time and in vitro analyses, we propose SON as a critical negative regulator of osteoclastogenesis. While basal SON expression maintains bone homeostasis by modulating NFATc1 and c-Fos, its pathological downregulation through aging or inflammation relieves this inhibitory brake, driving osteoclast overactivation and subsequent bone loss. Then, a ceRNA network was integrated to identify five related miRNAs, including hsa-miR-548n, hsa-miR-3163, hsa-miR-3143, hsa-miR-3119, and hsa-miR-302c-5p. miR-548n is known to regulate osteopontin (OPN) in OA [26], while miR-3163 is primarily associated with cancer and the NF-kB pathway [27]. This prediction further indicates that microRNAs (miRNAs) and long non-coding RNAs (lncRNAs) in OP and OA comorbidity offer new potential research targets [28,29,30,31]. This indicates that SON, as an RNA splicing and epigenetic regulatory factor [16,32], can play a protective role in preventing the occurrence of osteoporosis and osteoarthritis.

To further evaluate the druggability of the SON protein and its potential for clinical application, we conducted a virtual screening of the FDA-approved drug database. Among these, the Nilotinib-SON complex demonstrates the most favorable binding energy (−9.70 kcal/mol), suggesting a highly stable interaction. Nilotinib, a drug developed for the treatment of leukemia in clinical practice, is a Bcr-Abl tyrosine kinase inhibitor with anti-tumor activity [33]. Moreover, our studies have reported the inhibitory effect of Nilotinib on osteoclast differentiation, which further demonstrates the effectiveness of the target and the drug. However, it should be noted that the effect of Nilotinib on osteoblasts is bidirectional [34,35]. Therefore, further verification is needed for the clinical application of this drug. These findings not only elucidate the molecular mechanism of SON in comorbid diseases but also provide important clues for developing therapies targeting both osteoporosis and osteoarthritis.

This study identified the crosstalk genes and identified gene co-expression modules in OP and OA, thereby providing new insights into the comorbidity mechanisms between OP and OA. Crucially, the expression of SON can inhibit the development of OP and OA. Notwithstanding its contributions, this study is constrained by several limitations. Factors such as population stratification, disease heterogeneity, and underlying genetic variances may account for the discrepant causal roles of NUP153 observed in OP and OA. And the current ceRNA network is derived exclusively from bioinformatic predictions, necessitating further empirical evidence to corroborate these computational findings. Furthermore, the constrained sample size introduces a potential risk of statistical bias, necessitating cautious interpretation of the findings. Future longitudinal studies with larger sample sizes are needed to validate our findings. Moreover, given that blood-based eQTLs may not faithfully mirror bone biology, incorporating bone-specific datasets will be essential for a more comprehensive understanding in the future. Although the roles of SON in OP and OA have been demonstrated, the deeper mechanisms involving mRNA surveillance pathways in the development of comorbidity remain unclear. Therefore, establishing a comorbidity model would enhance the credibility of our findings.

## 4. Methods

### 4.1. Study Design

This study was conducted in three phases. First, shared molecular mechanism underlying OP and OA was explored in WGCNA. We focused on large-sample datasets GSE56815 and GSE48556 for the training group and selected GSE7429 and GSE12021 as verification groups for DEG analysis, ensuring sample type consistency.

Following the exploratory analysis, we prioritized hub genes with high intramodular connectivity and significant pathological relevance to both OP and OA for subsequent functional validation. A two-sample MR analysis was performed using pooled data from GWAS, treating gene expression in blood as the exposure and disease onset as the outcome. To better pinpoint hub gene function, we analyzed femoral head sequencing data from patients with OP and OA using single-cell sequencing data analysis, identifying hub genes expression within specific cell clusters. Afterwards, we predicted the mRNA-miRNA-lncRNA regulatory network for the identified hub genes, elucidating associated molecular mechanisms.

**Figure 7:**
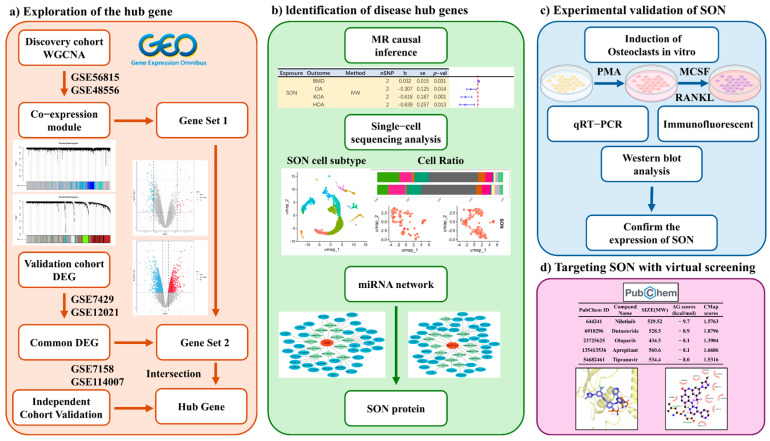
Overall study design including bioinformatics screening, disease association identification, experimental validation and drug virtual screening. (**a**) Bioinformatics screening workflow for hub gene identification. GEO, Gene Expression Omnibus; WGCNA, weighted gene co-expression network analysis; DEG, differentially expressed gene; Hub Gene, key regulatory gene. (**b**) Disease association identification of SON via multi-omics analysis. MR, Mendelian randomization; SNP, single nucleotide polymorphism; BMD, bone mineral density; OA, osteoarthritis; KOA, knee osteoarthritis; miRNA, microRNA. (**c**) In vitro experimental validation of SON expression. PMA, Phorbol 12-myristate 13-acetate; MCSF, macrophage colony-stimulating factor; RANKL, receptor activator of nuclear factor-κB ligand; qRT-PCR, quantitative real-time polymerase chain reaction. (**d**) Virtual screening of SON-targeting compounds.

Finally, we further investigated the expression and function of hub gene through qRT-PCR, WB and IF. To explore the druggability of the target protein, we conducted a virtual screening using the FDA database and verified the initial efficacy of the drug. The design of our study scheme is shown in Figure 7.

### 4.2. Data Source and Study Population

For gene expression analysis, data on differentially expressed genes (DEG) and weighted gene co-expression network analysis (WGCNA) were downloaded from the NCBI GEO public database (https://www.ncbi.nlm.nih.gov/). For robust WGCNA, only datasets with sample sizes >20 were retained from the initially retrieved29 OP-related and 35 OA-related datasets. And peripheral blood samples were prioritized to maintain consistency across datasets and to provide a holistic view of the integrated disease profiles. Further methodological specifications are delineated in Table 1. GSE56815 contains data from 40 White females with High BMD and 40 with Low BMD at the hip [36]. We used GSE7429 [37] as a validation cohort and performed principal component analysis (PCA) to cluster the groups, selecting three representative samples from each group for differential analysis. GSE48556 was used as a discovery cohort for osteoarthritis [38], including 106 OA patients and 33 normal controls, whereas GSE12021 served as a validation cohort to further investigate the molecular mechanisms of OA [39]. The stability of *SON* was further validated in independent cohorts (GSE7158 and GSE114007), ensuring the reproducibility of our conclusions across geographically and demographically distinct populations [40,41].

To obtain the instrumental variables of MR analysis, we retrieved eQTLs genetic data for the selected genes from the IEU OpenGWAS project website, using the corresponding Ensembl IDs as the GWAS identifiers. Genetic data for osteoarthritis were obtained from the GWAS catalog (https://www.ebi.ac.uk/gwas/, accessed on 30 April 2026), with the IDs GCST005814, GCST005813, and GCST005810 [42]. Genetic data for BDM were derived from the IEU OpenGWAS project. Detailed information on the datasets used for MR analysis is provided in Table 2. Additionally, single-cell sequencing data from GSE169396 included femoral head samples from three patients with co-occurring OP and OA and one control patient without OP [43]. All datasets used in our study are publicly available, published in public databases, and have received ethical approval and informed consent. Therefore, no additional ethical approval is required.

### 4.3. Screening and Identification of Shared Genes in OP and OA

#### 4.3.1. WGCNA for Co-Expression Network Construction

The Low BMD datasets and OA datasets were systematically analyzed using WGCNA to investigate the relationship between gene networks and phenotype [44]. We constructed a co-expression network for all genes using the WGCNA-R package (v1.73), which identified the 10,000 most significantly variable genes for further analysis [45,46]. We set the minimum module size at 30 genes and calculated network topology with a soft-thresholding power ranging from 1 to 20, with optimal soft thresholds of 4 and 8 for the two datasets, respectively (Figure S7). To assess network connectivity, the weighted adjacency matrix was transformed into a topological overlap matrix (TOM), and a cluster dendrogram was generated using hierarchical clustering. Each branch in the dendrogram represented a module, with distinct colors assigned to each module. Genes were clustered into modules based on similar expression patterns and functional relevance. Genes from the modules showing the strongest correlation with disease were selected (r > 0.6 and *p* < 0.05). Co-expressed genes (Gene Set 1, GS1) were identified using the VennDiagram-R package (v1.7.3), and a Venn diagram was generated for visualization [47].

#### 4.3.2. Validation of Shared Gene Signatures Through DEGs Analysis

We used principal component analysis (PCA) dimensionality reduction to perform cluster analysis on the GSE7429 and GSE12021 datasets, each containing three samples. The validation set is only used for the characteristic observation of the disease and is not used for the main statistical inference. Differential expression analysis of datasets GSE7429, GSE12021, GSE7158, and GSE114007 were performed using limma-R package (v3.56.2), with screening criteria set at *p* < 0.05 and |log_2_FC| > 0.58 (corresponding to a 1.5-fold change). Genes meeting these criteria were considered DEGs. The pheatmap-R (v1.0.12) and ggplot2-R packages (v3.5.1) were used to create volcano plots and heatmaps to display differential gene expression patterns. Ultimately, the two sets of DEGs were combined into Gene Set 2 using a Venn diagram.

### 4.4. Multimethod Verification of Target Genes and Construction of Regulatory Networks

#### 4.4.1. Selection of Instrumental Variables

To ensure the reliability of MR analysis, we followed several steps to obtain genetic instrumental variables (IVs) that conform to the hypothesis of MR. First, independent SNPs significantly associated with the gene’s eQTL (*p*-value < 1 × 10^−6^) were selected. Second, a clumping procedure was applied to exclude SNPs in strong linkage disequilibrium (LD), using European ancestry samples from the 1000 Genomes Project (R^2^ < 0.001, window size = 10,000 kb). Finally, the F-statistic was calculated to assess the strength of each SNP (F = beta^2^/se^2^) [48], with SNPs having an F-statistic greater than 10 considered strong enough to minimize potential bias. Before conducting the MR analysis, we ensured that the SNP effects on both exposure and outcome corresponded to the same alleles. Then we used the IEU OpenGWAS database for a phenotypic scan (https://gwas.mrcieu.ac.uk/) to confirm the independence of the IVs for MR.

#### 4.4.2. MR Analysis

The two-sample MR analysis was performed using the “TwoSampleMR” package (v0.6.14) in R version 4.3.1. The primary method was inverse-variance weighting (IVW) regression [49]. In cases where only one SNP was available, the Wald ratio was used as an alternative to IVW. Causal estimates were presented as odds ratios (ORs) with 95% confidence intervals (CIs). Heterogeneity was assessed using Cochrane’s Q statistic. If heterogeneity was significant (*p* < 0.05), the random effects model was used; otherwise, a fixed effects model was used. The MR-Egger method was applied to assess horizontal pleiotropy in the selected IVs. The robustness and consistency of the results were further evaluated using a leave-one-out analysis [50]. These sensitivity analyses ensure that the assumptions of MR, particularly the exclusion restriction assumption, are not violated.

#### 4.4.3. Single-Cell Sequencing Analysis

To delineate cell-type-specific *SON* expression, publicly available human bone single-cell RNA sequencing datasets from patients (GEO: GSM5201884) and healthy controls (GEO: GSM5201883) were analyzed. Quality control was performed to retain high-quality cells with 100 < nFeature_RNA < 6000 and percent.mt < 10%. Subsequent data processing was conducted using the Seurat-R package (v5.1.0), and the spatial relationships among clusters were visualized via UMAP [51,52]. The FindAllMarkers function in Seurat was used to identify markers for each cell type, and these cell markers were cross-referenced with the Cell Taxonomy database (https://ngdc.cncb.ac.cn/celltaxonomy/, accessed on 30 April 2026) and relevant literature. Manual adjustments were made as needed to ensure reliable annotation. The selected gene sets were mapped to the identified cell clusters, and differences in gene expression were analyzed across groups. Finally, we extracted the monocyte subpopulations and used Monocle to analyze the developmental trajectories, thereby providing insights into the lineage relationships and the biological processes related to the data.

#### 4.4.4. Construction of miRNAs-Target Gene Networks

To further explore the regulatory network related to the identified genes, we predicted downstream miRNAs using miRanda (please note that the microRNA.org website is no longer in service; for details of the algorithm, please refer to PMID: 18158296), TargetScan (http://www.targetscan.org/), and miRDB (http://mirdb.org/) [53]. We then predicted miRNA-lncRNA interactions using spongeScan. The regulatory network was visualized using Cytoscape software (version 3.10.1).

### 4.5. In Vitro Verification

#### 4.5.1. Reagents and Media

RPMI-1640 medium and fetal bovine serum (FBS) were supplied by Gibco (Thermo Fisher Scientific, Waltham, MA, USA). PMA originated from MedChemexpress. rhM-CSF and rhRANKL were purchased from R&D Systems (Minneapolis, MN, USA). Anti-β-actin (AC006) and anti-CTSK (A1782) were purchased from ABclonal (Wuhan, China). Anti-SON (83787-5-RR) and anti-NFATc1 (66963-1-Ig) were from Proteintech (Chicago, IL, USA). The corresponding secondary antibodies were purchased from Beyotime (Shanghai, China), and DAPI staining solution was bought from Beyotime (Shanghai, China). And MedChemExpress (Monmouth Junction, NJ, USA) provided the high-purity (≥99.9%) Nilotinib used in this study.

#### 4.5.2. Cell Culture and Osteoclast Differentiation

The THP-1 (Human Acute Monocytic Leukemia Cells) line was provided by the Cell Bank of the Chinese Academy of Sciences (Shanghai, China) and cultivated in RPMI 1640 medium supplemented with 10% FBS under 5% CO_2_ at 37 °C. For the in vitro osteoclast differentiation experiment, THP-1 cells were seeded at a density of 1 × 10^6^ cells per well in a 6-well plate and treated with 100 ng/mL PMA for 3 days. Once all the cells had adhered to the plate, rhM-CSF (25 ng/mL; R&D Systems, Cat# 216-MC) and rhRANKL (50 ng/mL; R&D Systems, Cat# 6449-TEC), were applied for 1, 3, and 5 days, respectively [54,55,56].

Bone marrow-derived macrophages (BMDMs) were isolated from the femur marrow of C57BL/6 mice (6–8 weeks old). After red blood cells were lysed, BMDMs were cultured in α-MEM supplemented with 10% fetal bovine serum (FBS) and 1% penicillin–streptomycin for 1 day. Nonadherent cells were collected and further cultured for 6 days in the presence of M-CSF (25 ng/mL; R&D Systems, Cat# 416-ML) and RANKL (50 ng/mL; R&D Systems, Cat# 462-TEC), with medium changes every 2 days to induce osteoclast differentiation.

#### 4.5.3. Quantitative Real-Time Polymerase Chain Reaction (qRT–PCR)

The total cellular RNA in different groups was obtained by Trizol reagent (Vazyme, r401-01, Shanghai, China). Then, with 1 μg of total RNA as the initial template, reverse transcription was implemented to synthesize complementary DNA (cDNA). qRT–PCR testing was carried out in a CFX96™ thermal cycler (Bio-Rad Laboratories, Hercules, CA, USA) by mixing cDNA proportionally with ChamQ SYBR qPCR Master Mix (Vazyme, Q321-02) and corresponding primers. The primer sequences of the target genes are listed in Table S13.

#### 4.5.4. Western Blot Analysis

Cellular proteins in the samples were lysed by using RIPA Lysis Buffer (Beyotime, P0013B) at predetermined time points after incubation. The total proteins were separated by 10% SDS-PAGE (EpiZyme Biotechnology, Cambridge, MA, USA), and at the end of electrophoresis the proteins were transferred onto nitrocellulose membranes. After closure with 5% skim milk or 5% bovine serum albumin (BSA) for at least 1 h, membranes were incubated separately with primary antibodies against β-Actin (1:1000), NFATc1 (1:2000), CTSK (1:500) and SON (1:2000) at 4 °C overnight. The corresponding secondary antibody dilutions (1:1000) were then added for 2 h at room temperature after washing membranes adequately with Tris-buffered saline Tween (TBST) for an appropriate time and frequency. Finally, to display the protein bands, the membranes were treated with enhanced chemiluminescence (ECL; Yeasen, Shanghai, China) reagents and imaged using Image Lab 3.0 software. Protein quantification (relative gray level of the bands) was then measured by ImageJ software (1.54g).

#### 4.5.5. Immunofluorescence Staining

Pre-cultured THP-1 cells were fixed with 4% PFA for 10 min and then blocked with QuickBlock™ Blocking Buffer for Immunol Staining (Beyotime, P0260) for 30 min. DAPI was incubated in 0.2% BSA-PBS for 5 min, followed by three rinses with PBS and photographed with a fluorescence microscope. After co-incubation with anti-NFATc1 primary antibody (1:300) and anti-SON (1:300) antibody at 4 °C for 16 h, cells were rinsed with PBS and incubated with the corresponding fluorescent secondary antibody (conjugated to Alexa Fluor 594, ab150080, Abcam and Alexa Fluor 488, ab150113, Abcam, Cambridge, UK) for 1 h under light-protected conditions. Visualization was achieved with a fluorescence microscope (Zeiss, Oberkochen, BW, Germany), and the mean fluorescence intensity was measured using ImageJ.

#### 4.5.6. Cytotoxicity Assay

THP-1 monocytes (1 × 10^4^ cells/well) or BMDMs (1.5 × 10^4^) were plated in 96-well plates, permitted to adhere overnight, and then exposed to graded doses of Nilotinib for 72 h. Afterward, cell viability was quantified with the Cell Counting Kit-8 (Yeasen, 40203ES76); following a 2 h incubation, absorbance at 450 nm was recorded on a BioTek microplate reader (Venuski, VT, USA) to assess drug cytotoxicity.

#### 4.5.7. Cellular Thermal Shift Assay (CETSA)

We centrifuged the extracted THP-1 cell proteins. The protein lysate was evenly distributed in the PCR tubes. Each tube was incubated with the drug or DMSO on ice for 30 min. Then, the samples were heated at different temperatures (40, 45, 50, 55, 60, 65) for 5 min. After centrifugation, the supernatant was collected and the SON protein level was detected by Western blotting.

#### 4.5.8. Tartrate-Resistant Acid Phosphatase (TRAP) Kit for Cell Culture

Following a wash with phosphate-buffered saline (PBS), cells were fixed in 4% paraformaldehyde (PFA; Biosharp, Hefei, China). Subsequently, TRAP staining was carried out in accordance with the manufacturer’s protocol, using a commercial kit (Amizona Scientific, Birmingham, AL, USA). Mature osteoclasts were defined as multinucleated cells (>3 nuclei). Finally, images of these cells were acquired using an inverted microscope (Zeiss, Germany) and analyzed with ImageJ software to quantify both the number and the surface area of osteoclasts.

### 4.6. Virtual Screening and Molecular Docking Visualization

We obtained information on 966 FDA-approved drugs from the PubChem database and then constructed the active site of the SON protein. Molecular docking was performed using SaiVina to evaluate the binding affinity of the drugs to the protein. The top 3% of drugs in terms of binding affinity were selected (binding energy: <−7.5 kcal/mol). To identify potential small-molecule therapeutics capable of alleviating the pathological condition, we utilized the Connectivity Map (CMap) L1000 platform to query gene sets selectively inhibited in GS2, prioritizing candidate compounds with a “norm_cs” > 1.5 and a “fdr_q_nlog10” > 1 for subsequent experimental validation. Further, ADMET was used to assess the pharmacokinetics of the drugs. The results were visualized using PyMOL (3.10.14) and LigPlus (2.2.4).

### 4.7. Statistical Analysis

All data were displayed as mean ± standard deviation (SD) and were analyzed with GraphPad Prism software version 10.0. Statistical significance was determined through one-way or two-way analysis of variance (ANOVA) with Tukey’s test, with *p* < 0.05 considered statistically significant (* *p* < 0.05, ** *p* < 0.01, *** *p* < 0.001).

## 5. Conclusions

This study revealed the dual role of the *SON* gene in OP and OA. This evidence suggests that *SON* expression may serve as a protective factor against the comorbid progression of OP and OA. Moreover, by discovering that SON acts as a potential therapeutic target for nilotinib, our study identifies a promising avenue for precision treatment in patients with comorbid osteoporosis and osteoarthritis, though further functional validation remains essential. However, these theories still require further verification through clinical experiments.

## Figures and Tables

**Table 1:** Data source and characteristic information of discovery cohort and validation cohort.

Group	GSE Number	Platforms	Sample	Disease	Origin	Race	Gender	Function	PMID
Discovery cohort	GSE56815	GPL96	80	Low BMD	Peripheral Blood	Caucasian	Female	WGCNA	29330445
Discovery cohort	GSE48556	GPL6947	139	Osteoarthritis	Peripheral Blood	Caucasian	Female	WGCNA	23864235
Validation cohort	GSE7429	GPL96	6	Low BMD	Peripheral Blood	Caucasian	Female	DEG	18433299
Validation cohort	GSE12021	GPL96	6	Osteoarthritis	Synovium	Caucasian	Female	DEG	18721452
Validation cohort	GSE7158	GPL570	10	Low PBM	Peripheral Blood	Asian	Female	Additional analysis	19223260
Validation cohort	GSE114007	GPL11154	12	Osteoarthritis	Cartilage	Caucasian	Male and Female	Additional analysis	30081074
Validation cohort	GSE169396	GPL11154	2	The comorbidity of OP and OA	Femoral head	Asian	Female	Single-cell RNA-seq	34111027

**Table 2:** Data sources and key information of Mendelian randomization analysis.

Study	Year	Population	Exposure	Sample Size	Number of SNPs
GCST005814	2018	European	OA	50,508	15,845,511
GCST005813	2018	European	KOA	22,347	15,708,690
GCST005810	2018	European	HOA	11,989	15,543,682
ebi-a-GCST90029004	2018	European	Heel BMD	583,314	11,972,309

## Data Availability

All data analyzed during this study were extracted by published sources listed and fully referenced.

## Supplementary Materials

The following supporting information can be downloaded at: https://www.mdpi.com/article/10.3390/ijms27114905/s1.
