# Supplementary material for: Targeting Osteoporosis-Osteoarthritis Comorbidity: Multi-Omics Identification of SON and Exploration of Therapeutic Agents
Source: Int J Mol Sci. 2026 May 28;27(11):4905. doi: 10.3390/ijms27114905 (PMC13256101; doi:10.3390/ijms27114905)
Supplement: Supplementary file 1 [file ijms-27-04905-s001.zip › Supplementary figure.pdf]

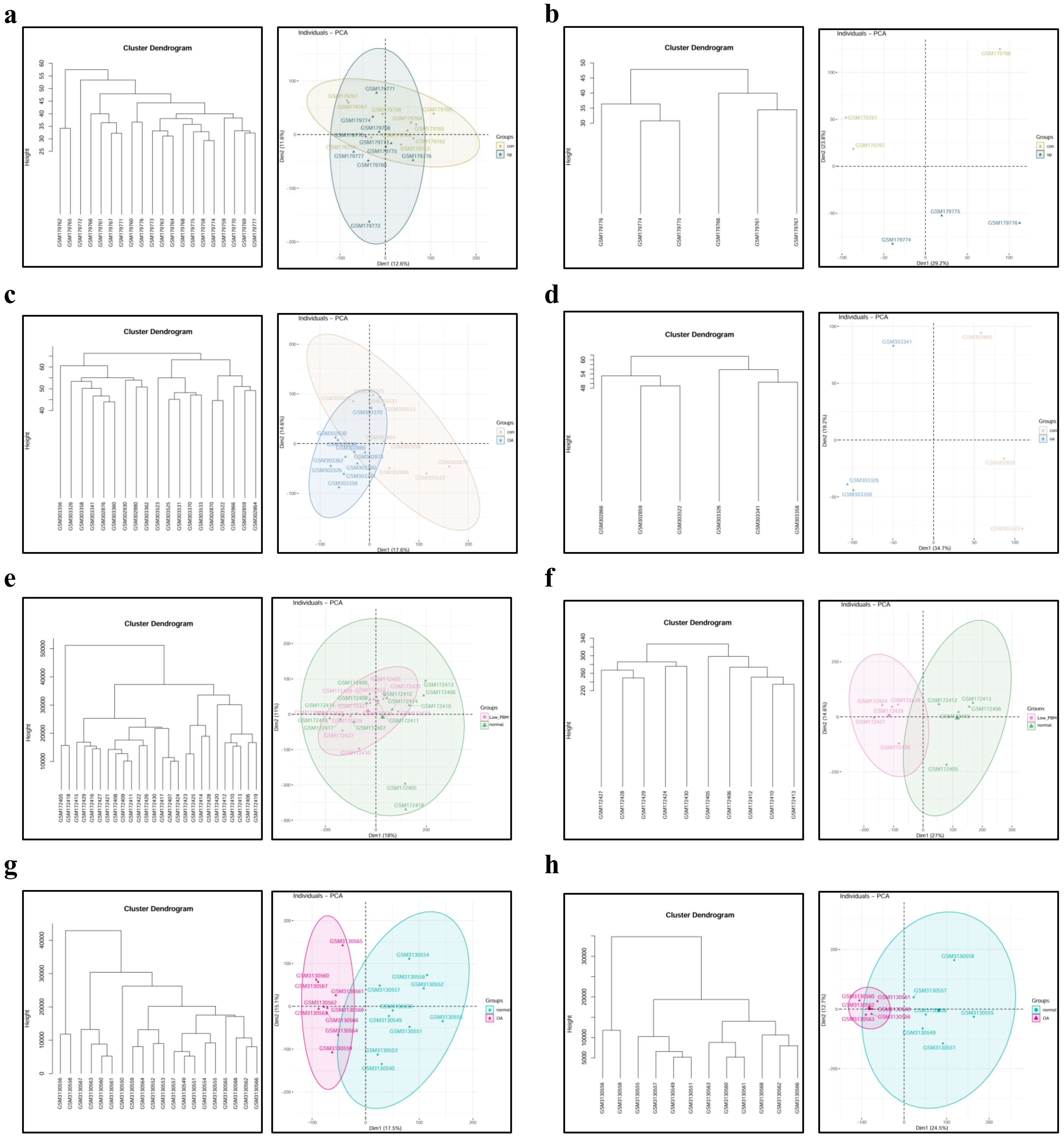

Figure S1. Cluster Analysis and Principal Component Analysis (PCA). (a) Cluster Analysis and PCA of the GSE7429 dataset ; (b) Cluster Analysis and PCA of the filtered GSE7429 dataset ; (c) Cluster Analysis and PCA of the GSE12021 dataset ; (d) Cluster Analysis and PCA of the filtered GSE12021 dataset ; (e) Cluster Analysis and PCA of the GSE7158 dataset ; (f) Cluster Analysis and PCA of the filtered GSE7158 dataset ; (g) Cluster Analysis and PCA of the GSE114007 dataset ; (h) Cluster Analysis and PCA of the filtered GSE114007 dataset .

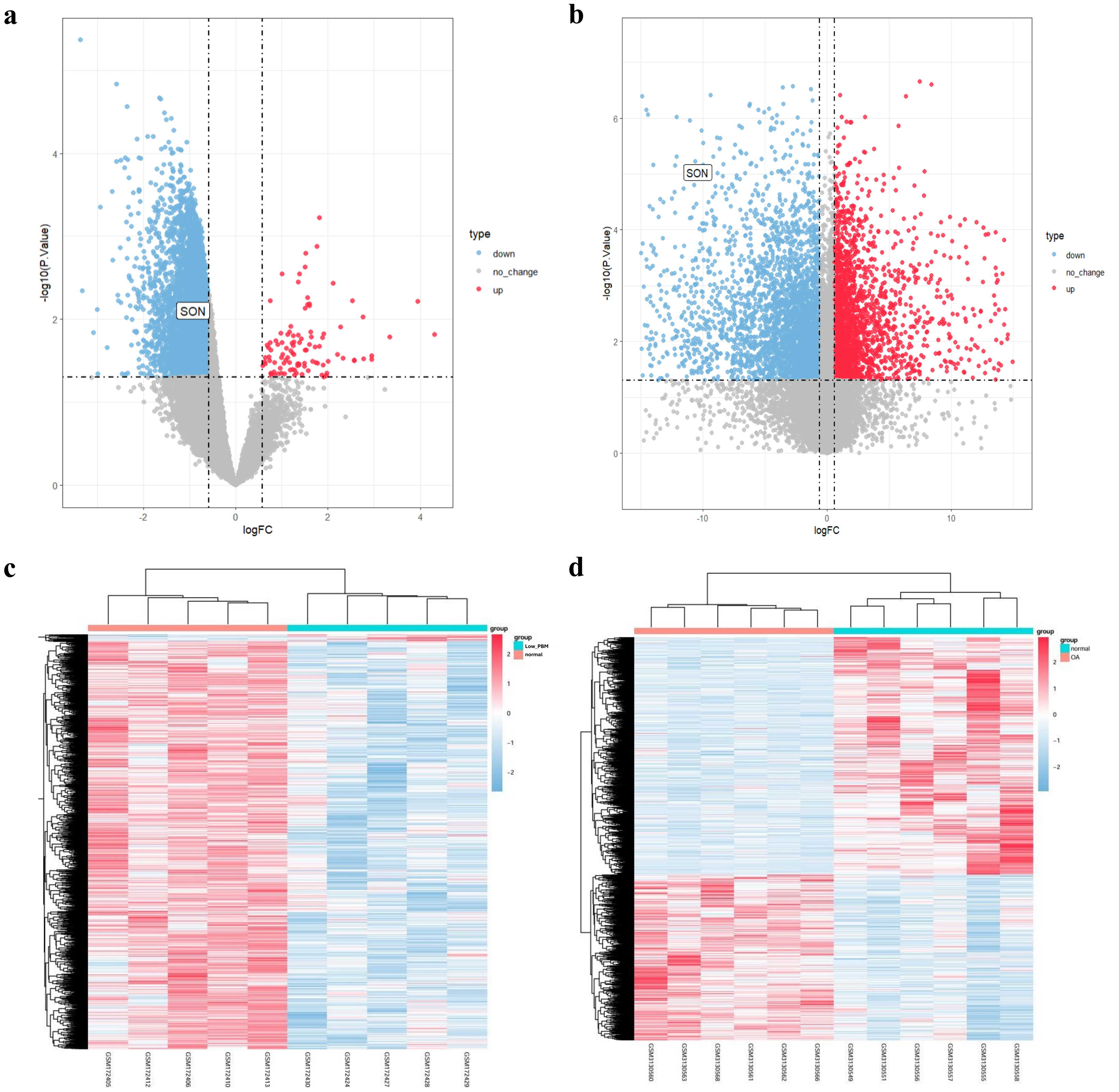

Figure S2. Volcano plot. (a) Volcano plot of GSE7158 ; (b) Volcano plot of GSE114007; (c) Heatmap of GSE7158; (d) Heatmap of GSE114007.

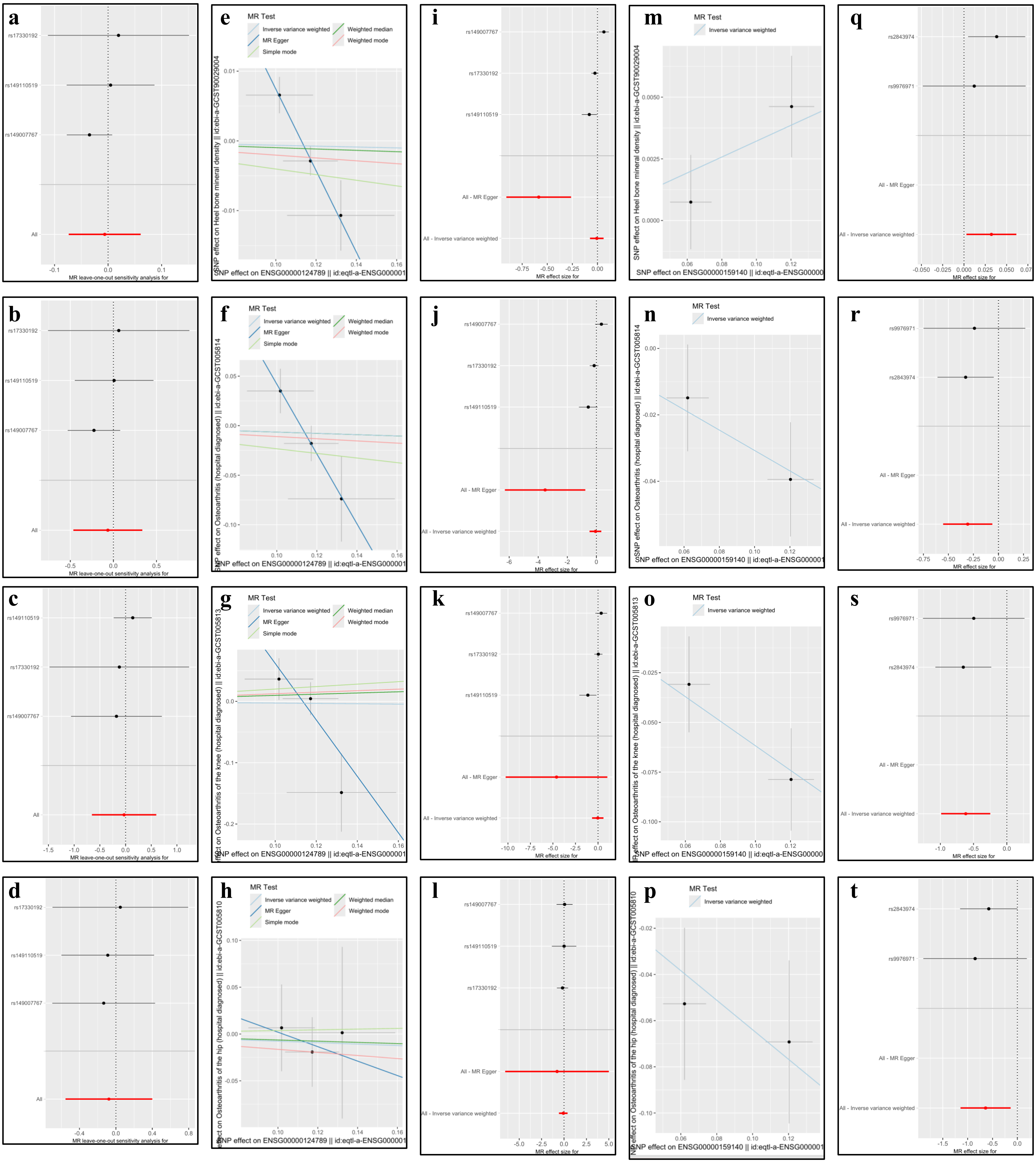

Figure S3. Sensitivity analyses for the causal effects of NUP153 and SON expression on bone-related traits. (a–d) Leave-one-out plots for the causal effect of *NUP153* on: (a) BMD, (b) OA, (c) KOA, and (d) HOA. (e–h) Scatter plots showing the associations between SNP effects on *NUP153* and their effects on: (e) BMD, (f) OA, (g) KOA, and (h) HOA. (i–l) Forest plots to assess horizontal pleiotropy for the effect of *NUP153* on: (i) BMD, (j) OA, (k) KOA, and (l) HOA. (m-p) Scatter plots showing the associations between SNP effects on *SON* and their effects on: (m) BMD, (n) OA, (o) KOA, and (p) HOA. (q–t) Forest plots to assess horizontal pleiotropy for the effect of *SON* on: (q) BMD, (r) OA, (s) KOA, and (t) HOA.

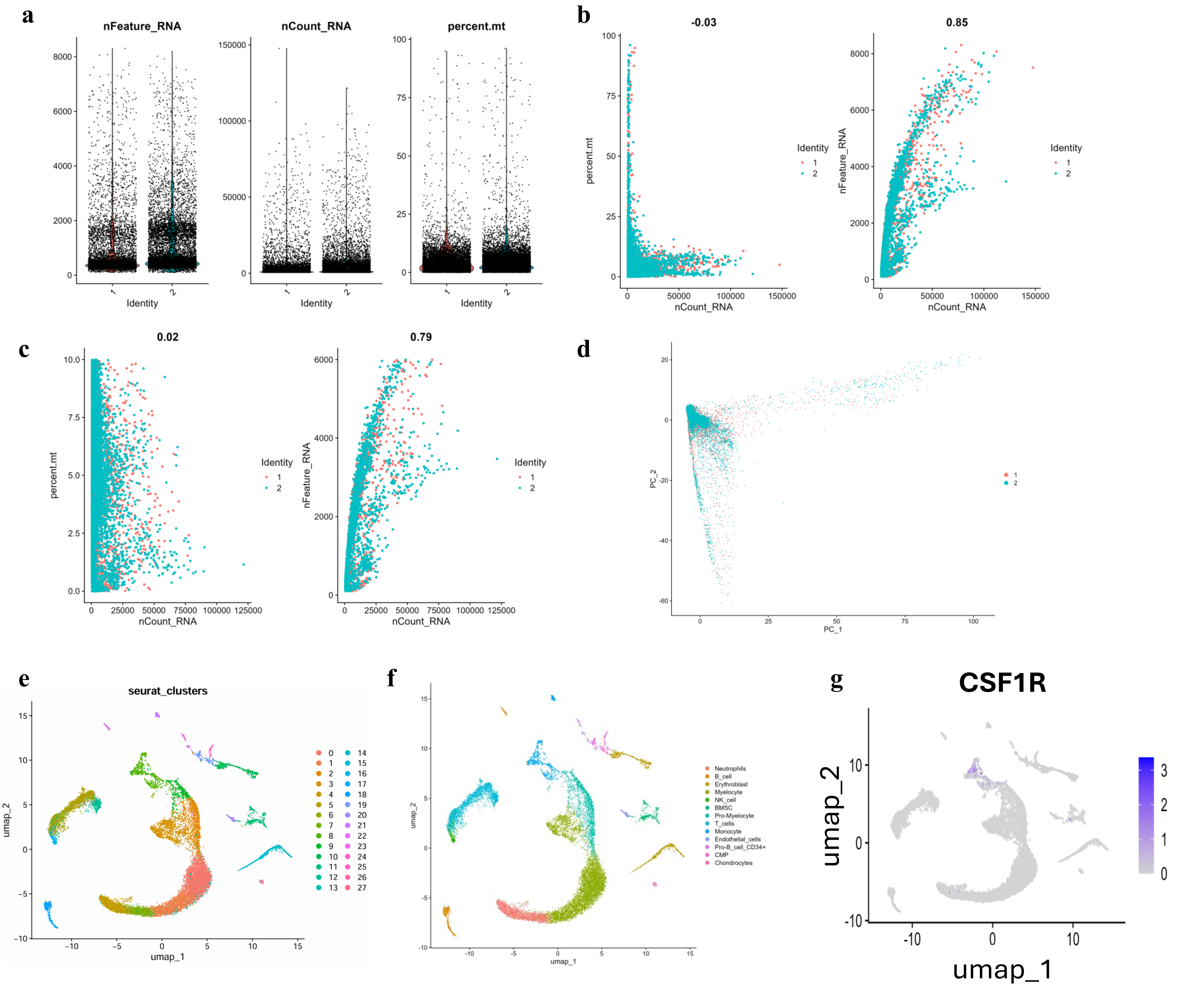

Figure S4. Quality control, dimensionality reduction, and cell-type annotation of single-cell RNA sequencing data. (a) Violin plots of quality control metrics. (b) Feature-feature relationship plots. (c)Post-filtering feature relationship plots. (d) PCA selection and distribution plot. (e) UMAP visualization of unsupervised clustering. (f) UMAP visualization of annotated cell types. (g) FeaturePlot of the lineage-specific marker CSF1R.

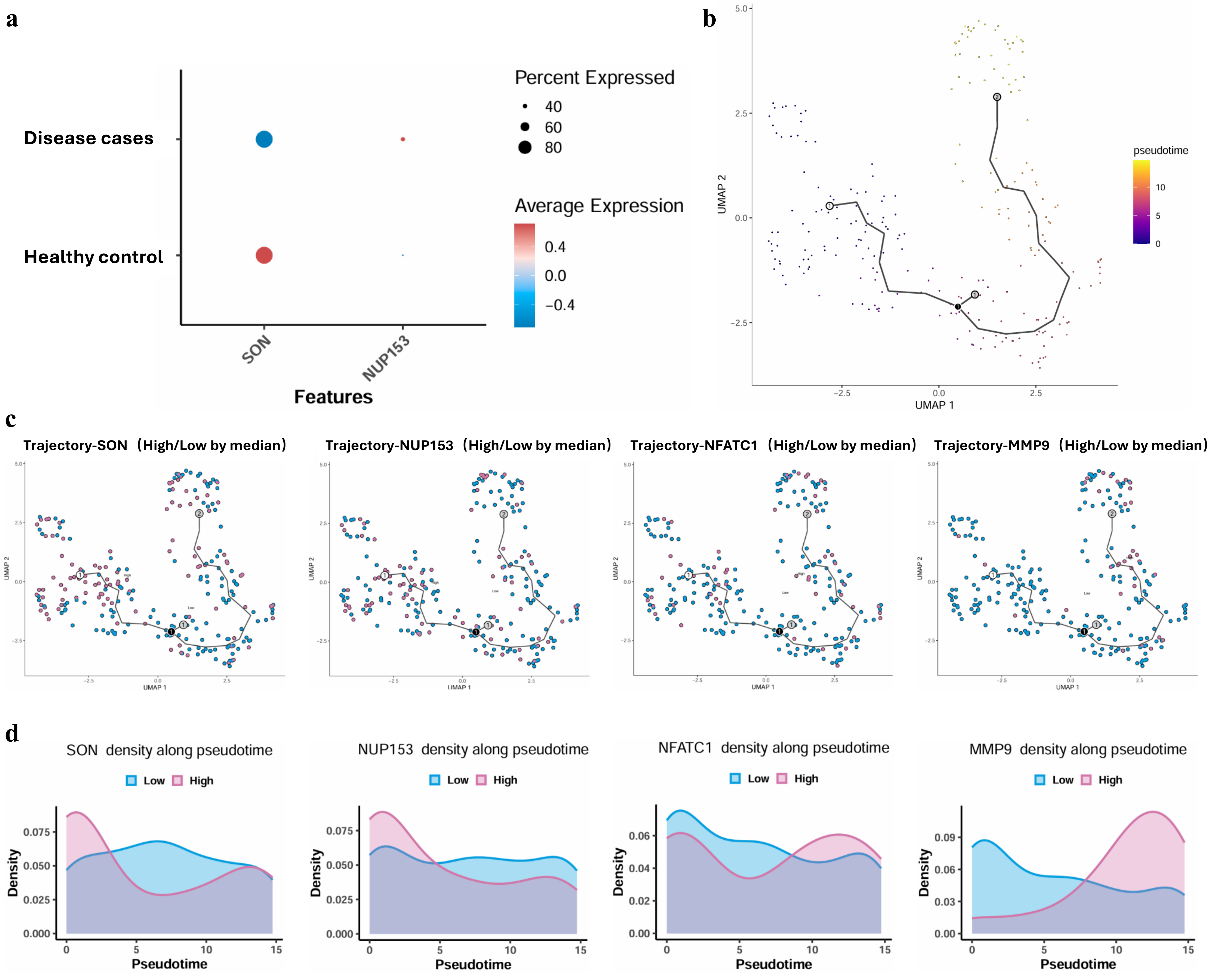

Figure S5. Analysis of the temporal trajectory of single-cell monocyte subpopulations.
(a) Differences in cell gene expression between the disease group and the healthy control group. (b) The temporal trajectory of monocyte subpopulations and the evolution trend of cell states during disease progression. (c) Changes in the expression of key genes in accordance with the proposed time sequence (SON, NUP153, NFATC1, MMP9). (d) Key gene expression characteristics of cells with high/low trajectory scores on the pseudo-time axis (SON, NUP153, NFATC1, MMP9).

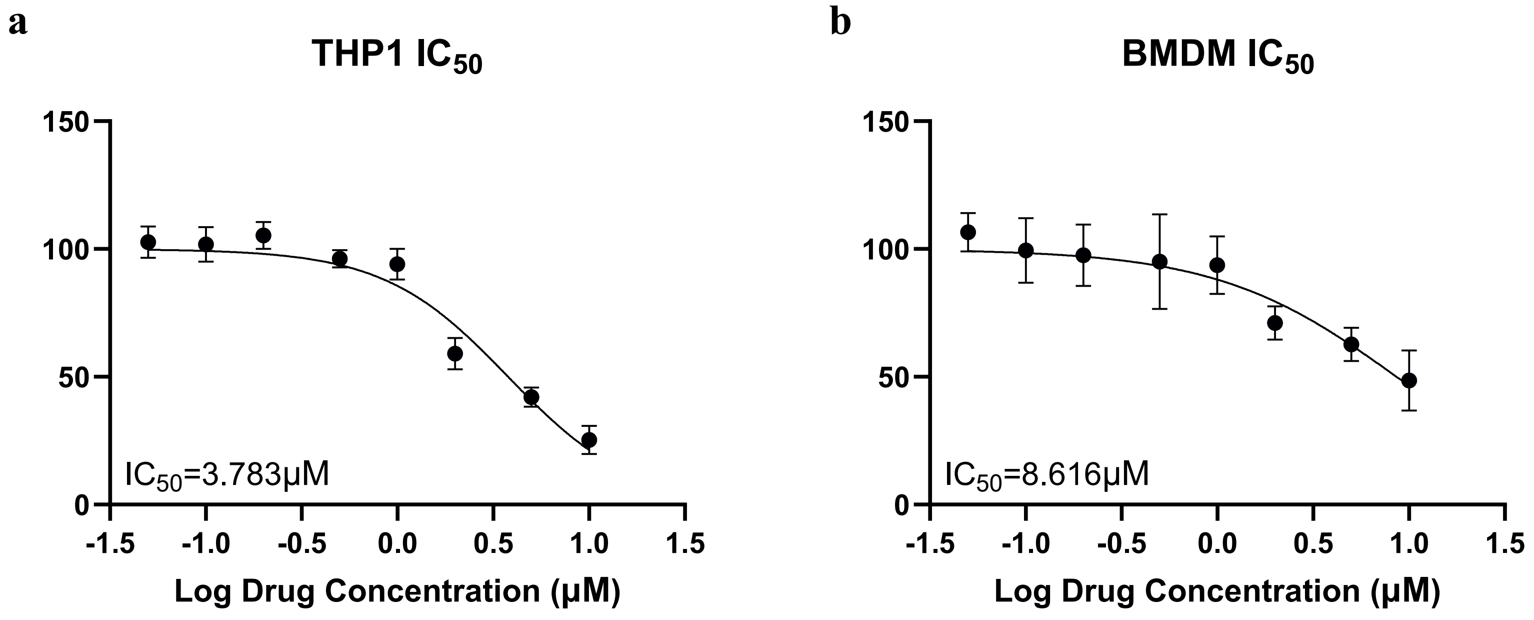

Figure S6. Dose-response curves of Nilotinib in THP-1 and BMDM cells. Cell viability was assessed by CCK-8. Data are presented as mean ± SD from three independent experiments (n=5). IC50 values were determined as 3.783 μM for THP-1 cells and 8.616 μM for BMDM cells using non-linear regression analysis.

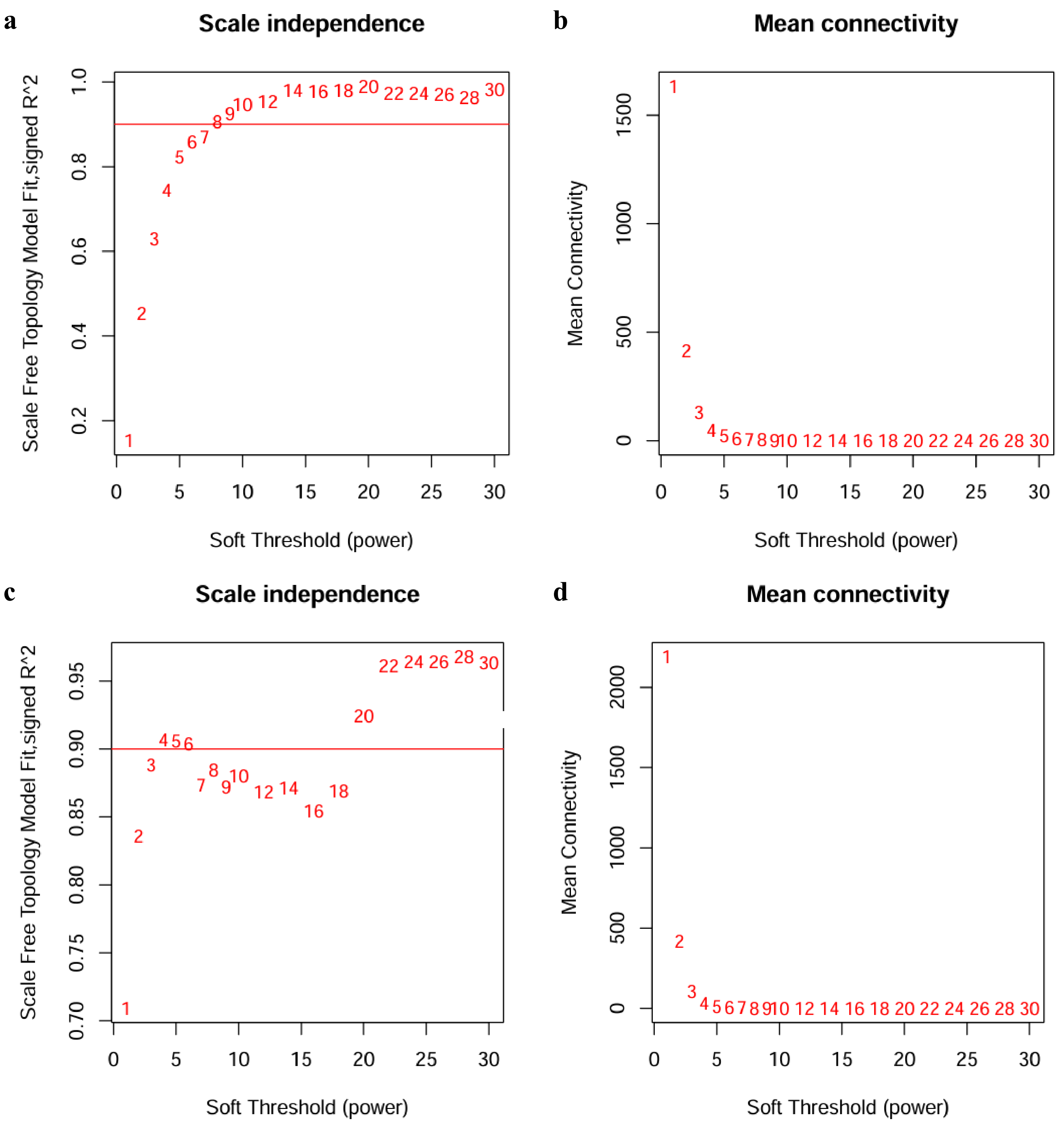

Figure S7. Determination of soft-thresholding power for weighted gene co-expression network construction. (a) Analysis of scale-free fit index for GSE56815; (b) Analysis of mean connectivity for GSE56815; (c) Analysis of scale-free fit index for GSE48556; (d) Analysis of mean connectivity for GSE48556.
